# Perinatal outcomes of severe preeclampsia/eclampsia and associated factors among mothers admitted in Amhara Region referral hospitals, North West Ethiopia, 2018

**DOI:** 10.1186/s13104-019-4161-z

**Published:** 2019-03-15

**Authors:** Misganaw Fikirie Melese, Marta Berta Badi, Getie Lake Aynalem

**Affiliations:** School of Midwifery, University of Gondar, Gondar, Ethiopia

**Keywords:** Perinatal, Outcomes, Severe preeclampsia and eclampsia, Ethiopia

## Abstract

**Objective:**

This study was aimed to assess perinatal outcomes of severe preeclampsia/eclampsia and associated factors among mothers admitted in Amhara Region referral hospitals, North West Ethiopia, 2018.

**Results:**

This study revealed that the overall prevalence of unfavorable perinatal outcome from the severe preeclamptic/eclamptic mothers was 46.5%. It shows that the finding of unfavorable perinatal outcome was high which needs an obligation to put our recommendation as improvement of health care set ups for preventions as well as interventions of such problems. Variables which were positively associated with unfavorable perinatal outcomes were: maternal educational status (AOR = 6.6, 95% CI 1.32, 10.03), parity (AOR = 8.3, 95% CI 6.27, 27.02), gestational age in weeks (AOR = 9.6, 95% CI 2, 18.65) and time of drug given for the mother (AOR = 3.8, 95% CI 1.81, 8.07).

## Introduction

Preeclampsia: is the new onset of hypertension and proteinuria after 20 weeks of gestation in a previously normotensive woman [1, 2]. Severe preeclampsia is a serious clinical type of preeclampsia manifested by at least one of the followings: persistent increase in blood pressure (≥ 160/110 mmHg), hepatic or renal failure, platelet count < 100,000/mm^3^, hemolytic elevated liver enzymes and low platelet count (HELLP) syndrome, cerebral or visual disturbances, persistent severe epigastric pain and pulmonary edema [3].

Eclampsia is a serious obstetric emergency with new onset of grand mal seizure during pregnancy or postpartum in a woman having signs or symptoms of preeclampsia [4–7].

The sequel of severe preeclampsia and eclampsia includes organ failure, loss of consciousness and finally loss of lives of both mother and fetus [4, 8, 9]. A systematic review for epidemiology of preeclampsia reviewed by USA investigators states that pregnancy induced hypertension remains a leading cause of maternal and perinatal morbidity and mortality worldwide [10]. Another study carried out in Mettu Karl referral hospital to assess maternal and perinatal outcomes of severe preeclampsia and eclampsia indicated that 12% of perinatal mortality [11]. The Government of Ethiopia in line with WHO takes a commitment to reduce perinatal mortalities through multiple high impact interventions at both facility and community levels to remove bottlenecks such as harmful traditional practices, poor infrastructure, shortage of transportation facilities and inadequate care at health facilities that can hamper access to safe motherhood services [12]. Even though, there were devotion to reduce pregnancy related complications and mortalities, perinatal deaths due to severe preeclampsia/eclampsia is still increasing worldwide [7, 13, 14].

According to different perspectives from different countries mentioned above showed that the burden of severe preeclampsia/eclampsia is not only limited to maternal health problem, but also significant number of fetal deaths [15–17].

Studies tried to investigate the trend, magnitude and associated factors of severe preeclampsia and eclampsia throughout the world [7, 14, 18–21], but almost all these studies used secondary data and limited to investigate feto-maternal outcomes.

This study, therefore, was conducted to determine the perinatal outcomes of severe preeclampsia and eclampsia by incorporating time of drug initiation in addition to the variables of the previous studies used.

## Main text

### Methods

#### Study design and setting

Institutional based cross-sectional study was conducted among 456 severe preeclamptic and eclamptic mothers from April 1 to September 30, 2018. The setting was in referral hospitals of Amhara Region, North West Ethiopia. This regional state has 67 hospitals among which 5 are referrals that is: University of Gondar Teaching Referral Hospital, Felege Hiwot Referral Hospital, Dessie referral hospital, Debremarkos referral hospital, and Debrebirhan referral hospital. Three of them were selected with a lottery method (Felege-Hiwot Referral Hospital, Debre-Markos Referral Hospital and Dessie Referral Hospital).

#### Sample size and sampling procedures

The sample size was determined by counting the 2017, severe preeclampsia and eclampsia cases for 6 months preceded the data collection time in the logbooks of randomly selected referral hospitals of Amhara Region. According to these logbooks, Felege-Hiwot Referral Hospital, Debre-Markos Referral Hospital and Dessie Referral Hospital have 174, 138 and 102 severe preeclamptic/eclamptic cases from October 1/2017 to March 30/2018 (the preceded 6 months of data collection) respectively. The sample size we used was the total cases of the three referral hospitals which was totaled as 414 we added a 10% expected non-response rate which became 456.

We used census to get enough sample size and we have gone with data collection until our sample size was saturated from each of the referral hospitals according to their case flows.

#### Operational definitions

##### Perinatal complication of severe preeclampsia

Complications of the newborn due to severe preeclampsia and eclampsia including low birth weight, still birth, intra uterine growth restriction, intra uterine fetal death, preterm birth, low APGAR score, birth asphyxia and abortus [2].

##### Favorable perinatal outcome

Newborns from severe preeclampic and eclampic mothers end up without perinatal complications of severe preeclampsia and eclampsia [11].

##### Unfavorable perinatal outcome

Newborn from severe preeclampic and eclampic mothers ended up with at least one complication of severe preeclampsia and eclampsia [11, 20].

##### Low APGAR score

Apgar score less than 7 in either or both 1st and 5th min of delivery.

#### Data collection procedures and tools

Data were collected using a semi-structured, pre-tested and interview based questionnaire adapted from the literatures. It was prepared in English and translated into local language Amharic and finally returned to English by English language expertise. Nine midwives were involved in the data collection process.

#### Data quality control

Semi-structured data collection tool was utilized and clarity of the tool was tested before the final utilization. The pretest was conducted among 5% of the sample size in Debre Berhan referral hospital. A 1 day training was given for data collectors and supervisors regarding the objectives of the study, data collection method and significance of the study. During data collection each data collector was supervised for any difficulties and directions and necessary corrections were provided.

#### Data processing and analysis

All collected data were rechecked for completeness, coded and entered using Epi Info 7.2, and exported to SPSS version 23 for cleaning and analysis. Bivariable logistic regression was employed to identify association, and multivariable logistic regression model was used to control the effect of confounders.

Variables having P-value less than 0.05 in the Bivariable analysis were fitted into the multivariable logistic regression model. 95% CI and odds ratio were computed and variables having P-value less than 0.05 in the multivariable logistic regression analysis were considered to declare statistical significance. Descriptive statistics like frequencies and cross tabulation was performed.

### Results

#### Socio-demographic characteristics

A total of 456 cases were included in the study among which, two hundred forty-three (53.3%) were severe preeclamptic and two hundred thirteen (46.7%) eclamptic.

The mean age of the participants was 28.3 years. As per educational status of the participants, 193 (42.3%) responded that they were unable to read/write. Most of the respondents, 429 (94.1%) were married. More than half of the participants, 235 (51.5%) were urban dwellers (Table 1).

**Table 1:** Socio-demographic characteristics among mothers with severe preeclampsia and eclampsia admitted, in Amhara region referral hospitals, North West Ethiopia, 2018 (n = 456)

Variables	Frequency	Percent
Maternal age		
Age ≤ 19	33	7.3
Age 20–35	349	76.5
Age ≥ 36	74	16.2
Religion		
Orthodox	347	76.1
Muslim	101	22.2
Protestant	7	1.5
Catholic	1	0.2
Marital status		
Married	429	94.1
Single	20	4.4
Divorced	4	0.9
Widowed	3	0.7
Educational status		
Unable to read and write	193	42.3
Able to read/write	38	8.3
Elementary	56	12.3
Secondary	71	15.6
College and above	98	21.5
Occupation		
Unemployed	16	3.5
Housewife	281	61.6
Private worker	76	16.7
Governmental worker	66	14.5
NGO	6	1.3
Student	11	2.4
Residence		
Urban	235	51.5
Rural	221	48.5
Traditional treatment use		
Yes	268	58.8
No	188	41.2
Monthly family income in USA dollar		
Income < 72.8 USA dollar	226	49.6
Income from 72.8 to 125.2 USA dollar	95	20.8
Income > 125.2 USA dollar	135	29.6

#### Obstetrical characteristics

Greater than half of the participants, 261 (57.2%) were Multiparas. The median gestational age of the respondents was 38 weeks, ranging from 21 to 43 weeks. Half of respondents, 225 (49.4%) gestational age was found to be between 37 and 40 weeks. More than two-third of the participants, 315 (69.1%) had ANC follow up. Three-fourth of the respondents, 346 (75.9%) had no history of abortion. Nearly two-third of participants, 292 (64%) with severe preeclampsia and eclampsia were unbooked in the referral hospitals of Amhara Region. Mode of deliveries were: − 172 (37.7%) vaginally after induction/augmentation, 136 (29.8%) SVD and 127 (27.9%) C/S.

#### Time of treatment and drugs given

Greater than half of the respondents, 267 (58.6%) had received medications lately (after they developed complications) where as 189 (41.4%) of them had got early (as soon as severe preeclampsia/eclampsia was diagnosed). Magnesium sulfate and Methyl dopa were the most frequently administered anti-convulsing and anti-hypertensive, drugs administered for 431 (94.5%) and 395 (86.6%) respondents respectively.

#### Perinatal outcomes of severe preeclampsia and eclampsia

Two hundred twelve (46.5%) newborns delivered from respondents were ended up with unfavorable outcomes of severe preeclampsia and eclampsia with 95% CI (42.2%, 51.1%) and nearly one-third of the fetuses, 103 (28.1%) were still births (Table 2).

**Table 2:** Cause of perinatal outcomes of severe preeclampsia and eclampsia among mothers admitted in Amhara Region referral hospitals, North West Ethiopia, 2018 (n = 456)

Fetal outcomes	Favorable	244 (53.5%)
	Unfavorable	212 (46.5%)

Cause of unfavorable outcomes	Frequency	Percent (%)
LBW	59	13
Low APGAR score	49	10.8
IUGR	20	4.4
Stillbirth	103	22
Preterm birth	49	10.8
Birth asphyxia	46	10.1
Abortus	41	8.9

#### Factors associated with the response variable

Bivariate analysis showed that: maternal educational status, monthly family income, maternal residence, parity, gestational age in weeks, maternal booking status and Time of drugs given were crudely associated.

Independently and positively associated variables in adjusted analysis were: maternal educational status, parity, gestational age in weeks and time of drugs given (Table 3).

**Table 3:** Binary and Multivariable logistic regression results of factors associated with perinatal outcomes of severe preeclampsia and eclampsia among women admitted in Amhara region referral hospitals, North West Ethiopia from April 1 to September 30, 2018 (n = 456)

Variables	Fetal outcome		COR (95% CI)	AOR (95% CI)
	Unfavorable	Favorable		
Educational status				
Unable to read and write	117	76	7.9 (4.29, 14.49)**	6.6 (1.32, 10.03)*
Read and write	20	17	6.0 (2.60, 13.96)**	4.6 (1.18, 12.01)*
Elementary school	32	26	6.3 (2.99, 13.28)**	4.3 (1.33, 13.63)*
Secondary school	27	43	3.2 (1.56, 6.61)	2.8 (0.89, 8.79)
College and above	16	82	1	1
Monthly family income				
Income < 72.8 USA dollar	140	86	8.4 (4.922, 14.20)	2.1 (0.84, 5.05)
Income from 72.8 to 125.2 USA dollar	50	45	5.7 (3.10, 10.49)	2.4 (0.86, 6.45)
Income > 125.2 USA dollar	22	113	1	1
Residence				
Rural	145	75	4.8 (3.25, 7.21)	1.3 (0.61, 2.70)
Urban	67	168	1	1
Parity				
Nully para	59	11	10.5 (5.27,21.07)**	8.3 (6.27, 27.02)*
Premipara	64	58	*2.2 (1.40, 3.36)* ^*^	5.2 (4.15, 13.97)*
Multi para	89	175	1	1
Gestational age in weeks				
GA 20–27	38	3	4.9 (1.38, 17.39)*	9.6 (2.00, 18.65)*
GA 28–36	76	7	4.2 (1.69, 10.40)*	5.4 (1.98, 14.73)*
GA 37–40	33	190	0.1 (0.03, 0.12)**	0.2 (0.08, 0.36)**
GA > 41	62	24	1	1
Booking status				
Unbooked	184	108	8.27 (5.16, 13.25)	1.84 (0.86, 3.93)
Booked	28	136	1	1
Time of drug given				
Late	178	89	9.1 (5.81, 14.29)**	3.8 (1.81, 8.07)**
Early	34	155	1	1

** P-value < 0.001,* P-value < 0.05, 1 = reference

## Discussion

This institutional based cross sectional study has attempted to assess the perinatal outcomes of severe pre-eclampsia/eclampsia and associated factors among women admitted in Amhara Region referral hospitals, North West Ethiopia, 2018.

The study results showed that the prevalence of unfavorable perinatal outcomes was 46.5% which is lower than a 5 years period cross sectional study in Addis Ababa town (66.4%). This difference might be due time gap as time goes, there is a likelihood of better health care system.

Stillbirth was one of the causes for unfavorable outcomes. 103 (22.6%) of newborns were stillbirths which is a higher finding compared with the studies done in Addis Ababa (8.5%) [14], Mettu Karl Referral Hospital (10.2%), Ghana (6.8%) [20] and Haiti (17.8%) [17] but lower than studies done in Nigeria (34.2%). It shows that stillbirth is still high which may alert policy makes, managers and health care providers to make health care set up better.

Forty-one (8.9%) of the pregnancies from severe preeclamptic/eclamptic mothers were ended up with abortion which is in line with a 3 years retrospective cross sectional study done on 121 cases in Mettu Karl Referral Hospital, Ethiopia (10.7%). This finding compared with Mettu Karl Referral Hospital has better implication because they used secondary data but ours is primary data, their study population was all hypertensive cases but ours is only severe preeclamptic/eclamptic cases.

LBW (16.1%), low APGAR score (13.4%), IUGR (5.4%), preterm birth (13.4%) and birth asphyxia (12.5%) were other causes of unfavorable perinatal outcomes of the study which each is lower than the studies done in Mettu Karl Referral Hospital (low birth weight of 30.5%, and low APGAR score of 18.5%, and preterm delivery 31.4%), Debre Berhan Referral Hospital, Ethiopia (39.4% low birth weight, 38.4% low APGAR score and 8.5% IUGR) and Iran (52.6% preterm, 9.9% IUGR and 17.3% LBW) [22]. The difference could be due to time gap.

One of the predictor variables for perinatal outcomes of severe preeclampsia–eclampsia was maternal educational status. Newborns from mothers who were with educational status of unable to read/write, able to read/write and elementary school were more likely to develop unfavorable perinatal outcomes compared with those mothers who were with educational status of college and above with the odds ratio of (AOR = 6.6, 95% CI 1.32, 10.03) (AOR = 4.6, 95% CI 1.18, 12.01) (AOR = 4.3, 95% CI 1.33, 13.63) respectively.

It shows that the higher the women`s educational status is, the lower the perinatal unfavorable outcomes of severe preeclampsia/eclampsia. It could be due to the fact that as educational status is good, the mothers are likely to improve their health care seeking behaviors including having adequate ante-natal care follow up which can improve the perinatal outcomes.

Parity was another predictor variable. Newborns delivered from nully and primi paras women were more likely to develop unfavorable perinatal outcomes when compared with multi paras women with the odds ratio of (AOR = 8.3, 95% CI 6.27, 27.02) and (AOR = 5.2, 95% CI 4.15, 13.97) respectively. This finding is in line with studies done in South East Nigeria [21]. It might be due to the fact that frequency and severity of preeclampsia–eclampsia is more common in nully paras and primi paras which may possibly affect the perinatal outcomes.

Gestational age was also associated with the outcome variable. Pregnancies interrupted from 20 to 27 and 28 to 36 completed weeks were with high unfavorable perinatal outcomes when compared with pregnancies terminated from 37–40 weeks with the odds ratio of (AOR = 9.6, 95% CI 2.00, 18.65) and (AOR = 5.4, 95% CI 1.98, 14.73) respectively. And when pregnancies terminated from 37 to 40 weeks compared with pregnancies terminated at 41 and above weeks, it shows 80% less likely to develop the unfavorable perinatal outcomes (AOR = 0.2, 95% CI 0.08, 0.36). It shows the favorable gestational age of pregnancy termination in severe preeclamptic-eclamptic mothers is from 37 to 40 weeks. These findings are supported by a study conducted in Mettu Karl Referral Hospital, Ethiopia [11]. This could be related with prematurity in early weeks and placental apoptosis in late weeks which both possibly affect the perinatal outcomes.

Additional variable that positively predicts the perinatal outcomes of severe preeclampsia–eclampsia was time of drugs given. Newborns delivered from severe preeclamptic–eclamptic mothers who were provided anti-convulsing and anti-hypertensive drugs lately were more likely to develop unfavorable perinatal outcomes than the mothers received these drugs early (AOR = 3.8, 95% CI 1.81, 8.07). It puts a recommendation of giving drugs early.

## Limitations

Long term perinatal complications were not addressed and we might be in fear of the nature of cross-sectional design.

## Abbreviations

ANC: ante natal care
HEELPS: hemolysis elevated liver enzymes and low platelet count syndrome
iugr: intra uterine growth restriction
SVD: spontaneous vaginal delivery
C/S: caesarean section
